# Biological Databases for Hematology Research

**DOI:** 10.1016/j.gpb.2016.10.004

**Published:** 2016-12-11

**Authors:** Qian Zhang, Nan Ding, Lu Zhang, Xuetong Zhao, Yadong Yang, Hongzhu Qu, Xiangdong Fang

**Affiliations:** 1 CAS Key Laboratory of Genome Sciences and Information, Beijing Institute of Genomics, Chinese Academy of Sciences, Beijing 100101, China; 2 University of Chinese Academy of Sciences, Beijing 100049, China

**Keywords:** Hematology, Hematological diseases, Omics data resources, Database, Bioinformatics

## Abstract

With the advances of genome-wide sequencing technologies and **bioinformatics** approaches, a large number of datasets of normal and malignant erythropoiesis have been generated and made public to researchers around the world. Collection and integration of these datasets greatly facilitate basic research and clinical diagnosis and treatment of blood disorders. Here we provide a brief introduction of the most popular **omics data resources** of normal and malignant hematopoiesis, including some integrated web tools, to help users get better equipped to perform common analyses. We hope this review will promote the awareness and facilitate the usage of public **database** resources in the **hematology** research.

## Introduction

Blood is incredibly important in providing oxygen, protecting from infection, and healing after injury. Disorders of blood system lead to different kinds of hematological diseases in millions of people every year globally. Blood cells consist of three types of cells, namely erythrocytes (red blood cells, RBCs), leukocytes (white blood cells), and thrombocytes (platelets), all of which are differentiated and developed from hematopoietic stem cells (HSCs). Erythropoiesis normally produces functional RBCs [1], whereas erroneous erythropoiesis would lead to anemia, leukemia, and other blood diseases [2].

Recent advances in next-generation sequencing (NGS) technologies have provided outstanding platforms in blood research. In particular, single-cell sequencing technology makes it feasible to trace the HSC specification, cell fate decision, and differentiation into various cell types at single-cell resolution [3], [4]. In addition, high-throughput sequencing also allows genome-wide analysis of transcription factor binding and histone modifications by chromatin immunoprecipitation sequencing (ChIP-seq) [5], identification of open regions of chromatin by DNase-Seq [5], as well as transcriptomic expression profiles by RNA-Seq [5]. Deeper understanding of the hematological processes of mammals has been driven by the development of these technologies [6]. Large organizations, such as the National Center for Biotechnology Information (NCBI), and projects collaborated by international research groups, for example the Encyclopedia of DNA Elements (ENCODE), and a variety of individual laboratories have produced and released many genome-wide datasets to public [7]. Thanks to the increasingly deeper interpretation of the human genome and the development of bioinformatics databases, we have now appreciated the human erythropoiesis more. Here we collect the most popular omics data resources of normal and malignant hematopoiesis (Table 1). These data components and some integrated web tools for common analyses are introduced in this review.

## European LeukemiaNet

Leukemia is a cancer of white blood cells with high incidence among all ages. To centralize the fragmented information of European leukemia, the European LeukemiaNet (ELN) was founded by the 6th Framework Program of the European Community in 2004 [8]. The website with friendly user interface delivers information about ongoing clinical trials to physicians and patients, as well as further information regarding leukemia research, such as via publishing study protocols. Meanwhile, ELN shares knowledge about study design and monitoring, as well as data management and analysis, and pushes forward the discussion on leukemia within Europe (http://www.leukemia-net.org/content/home/index_eng.html). As many as 17 work packages work separately on information integration about research, diagnosis, and treatment of leukemia. Furthermore, ELN also provides information for patients and physicians to better understand the leukemia, the diagnostic methods, and different therapies available.

## Red Cell Membrane Disorder Mutations Database

Red cell membrane inherited disorders involves either altered membrane structural organization or altered membrane transport function [9]. The Red Cell Membrane Disorder Mutations Database (http://research.nhgri.nih.gov/RBCmembrane/) contains the mutations associated with three major inherited blood disorders, namely hereditary spherocytosis, elliptocytosis, and pyropoikilocytosis, all of which are caused by the disorder of red cell membrane structural organization. The welcome page introduces the gene mutations associated with the three diseases, as well as the term linkages to the Online Mendelian Inheritance in Man (OMIM) database for related genes. This database provides detailed information of gene mutations occurring in one or more diseases in its submenu. In other submenus, users can also obtain additional detailed information about clinical research program and genetic counseling from the National Human Genome Research Institute (NHGRI), the United States. In addition, the submenus also provide the linkage to the University of California Santa Cruz (UCSC) database for some mutation genes. At the bottom of the menu, researchers can find the contact information if they have additions, updates, or descriptions of new mutations.

## dbRBC

The dbRBC database is one of the NCBI database resources that provides an integrated and freely-accessible platform for DNA sequencing data and clinical data associated with the human RBCs (http://www.ncbi.nlm.nih.gov/projects/gv/rbc/main.fcgi?cmd=init). It integrates the data from the Blood Group Antigen Gene Mutation Database (BGMUT) that records variations in genes encoding antigens for human blood groups from the NCBI [10]. Users could obtain the data from the download menu that directly links to the page of file transfer protocol. dbRBC homepage also offers the linkage to the parallel resources, such as dbMHC for data related to the human major histocompatibility complex (MHC) and dbLRC for resource available for human leukocyte receptor complex (LRC). These 3 public resources make up the database cluster for routine clinical applications [11], such as the ABO genotyping technology. Some additional practical tools are also provided, such as the Alignment Viewer and Primer Resource.

## CODEX

CODEX (http://codex.stemcells.cam.ac.uk/) is a database of mouse and human NGS experiments. The aim of CODEX is to provide an open-resource of NGS experiments processed by uniform procedures. In this database, metadata of human and mouse samples from hematological experiments are collected and sequencing data are uniformly processed and vetted [12]. CODEX also provides access to processed and curated NGS experiments, including ChIP-seq, RNA-seq, and DNase-seq. The main data sources of CODEX are NGS repositories, for instance, the Gene Expression Omnibus (GEO) and ArrayExpress. Besides, CODEX also provides a private site hosting non-published data. Furthermore, processed datasets can be analyzed online or downloaded. CODEX now covers data on 133 hematopoietic cells and embryonic stem cells, and 269 factors associated with these cells.

## The Erythron Database

The Erythron Database (ErythronDB; http://www.cbil.upenn.edu/ErythronDB) was built to facilitate access to erythroid expression data and the analysis results in murine primitive and definitive erythroid cells [13]. ErythronDB allows users to identify differentially-expressed genes and custom-made downstream analysis in the strategy module. Users are also permitted to save and share strategies with other registered users. The database integrates global gene expression profile data of primitive, fetal liver definitive, and adult bone marrow definitive erythroid using Affymetrix array for each maturation stage. ErythronDB supports complex investigations on expression parameters, as well as the Gene Ontology (GO) and the Kyoto Encyclopedia of Genes and Genomes (KEGG) annotations. To ensure abundant knowledge on mouse genes, ErythronDB displays links to external databases, including the Mouse Genome Informatics (MGI).

## Hembase

Hembase (http://hembase.niddk.nih.gov) provides genome-based access to human genes transcribed during erythropoiesis. By sequencing several thousand expressed sequence tags (ESTs) of human erythroid cells, including progenitor cells, precursor cells, and mature RBCs, the Hembase integrated these data to provide users a friendly browser and the genome portal. To date, the database contained 15,752 entries of ESTs and 380 genes associated with erythropoiesis [1]. Hembase provides cytogenetic band position as well as a unique name as concise annotations for each search entry. Users can search by gene name, keywords, or cytogenetic location. All the sequencing information in Hembase can be used without registration, and all ESTs can be downloaded from the NCBI UniGene Library Browser [14].

## BloodSpot

BloodSpot (http://www.bloodspot.eu) is a database including gene expression profiles of healthy and malignant hematopoiesis in humans or mice, which had been generated by oligonucleotide microarray chips and RNA sequencing [15]. This platform is an improvement and expansion of HemaExplorer and encompasses more than 5000 samples in total [16]. For each query gene or gene signature, BloodSpot provides three concomitant levels of visualization—gene expression, survival plot, and hierarchical tree of samples. Besides, BloodSpot also contains other built-in tools such as exploring the top correlated genes and calculating the student *t*-test significance between pairs of populations in the default expression plot. Another feature of BloodSpot is BloodPool, an assembled and integrated database collecting the results of multiple studies with more than 2000 samples focusing on acute myeloid leukemia (AML).

## BloodChIP

The BloodChIP database (http://www.med.unsw.edu.au/CRCWeb.nsf/page/BloodChIP) provides a user-friendly exploration and visualization of transcription factor (TF) binding sites in human CD34^+^ and leukemia cells produced by TF ChIP-Seq platform [17]. Users can enter the keywords about specific gene(s) or genomic region(s) to retrieve TF binding profiles. Users can also search all the target genes for a combination of selected TFs or for any selected TFs in specific cell type(s). Currently, BloodChIP covers data on four cell types, *i.e.*, CD34^+^ hematopoietic stem and progenitor cells (HSPCs), megakaryocytes, SKNO-1, and K562. To maximize the utility of these data, this database has been integrated with many public data for insights into the transcriptional regulation of query genes, such as gene expression data, histone ChIP-seq data, and DNase-seq data from the Human Epigenome Atlas and ENCODE database [7], [18].

## Leukemia Gene Atlas

Leukemia Gene Atlas (LGA) database is a public platform integrating diverse genomic data published in the leukemia field [19]. The LGA supports comprehensive research, analysis, and browse functions for more than 5800 leukemia and hematopoiesis samples sequenced by multiple platforms, such as microarray, DNA methylation, SNP, and other high-throughput sequencing manners. The database contains information on studies from various aspects, such as prediction of molecular subtypes of leukemia, human hematopoiesis, and TF binding sites imported from the GEO. LGA also has established quality control procedure to filter out qualified data imported from other datasets. Results of each study include differentially-expressed genes, GO annotations, copy number alterations, and an extract of the Catalogue of Somatic Mutations in Cancer (COSMIC) database. The LGA database is freely accessible at http://www.leukemia-gene-atlas.org/LGAtlas/.

## Diamond-Blackfan anemia mutation database

Diamond-Blackfan anemia (DBA) is a hereditary bone marrow failure syndrome characterized by the marked heterogeneity of clinical symptom, such as anemia, developmental abnormalities, and an increased risk of malignancy [20], [21], [22]. The DBA mutation database was built aimed to help researchers and physicians to better understand the mutations found in patients. This database is based on the Leiden Open Variation Database (LOVD) system (http://www.dbagenes.unito.it). The database comprises of 27 published mutations in *RPS11* gene, the main contributor to DBA. Each mutation is described in detail with both tables and graphs, including gene information, sequence information, and graphic displays from UCSC [23], [24]. The database provides information on changes in DNA, RNA, and protein, as well as the frequency of the mutations via a convenient search interface. Users are welcome to submit mutations after they register as a submitter.

## Concluding remarks

**Figure 1:**
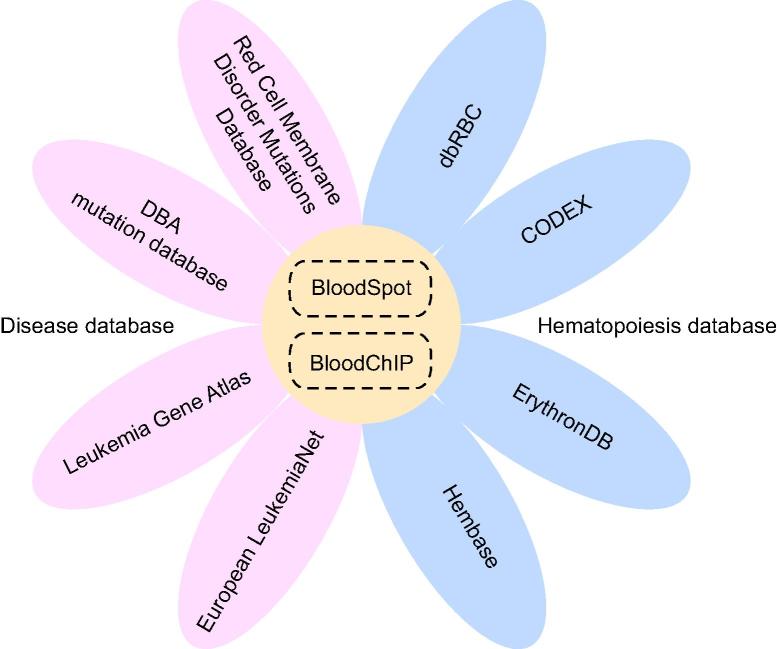
**Integrated figure of database in the fields of hematopoiesis and blood diseases** The 10 database mentioned in the current review are classified into 3 categories. Four databases marked with red petals on the left side of the flower are disease databases, providing biological data of hematopoietic disorders. Another four databases marked with blue petals on the right side of the flower are hematopoiesis database, providing information on normal hematopoietic development. The remaining two databases marked in yellow in the center of the flower are integrated databases. DBA, Diamond-Blackfan anemia; ErythronDB, the Erythron Database.

Abnormal development of blood cells has been widely studied in the past several decades. Due to the recent technological advances, a large amount of data for erythrocyte differentiation has been generated, producing valuable resources for understanding pathogenesis. This review offers a brief introduction of multiple databases in the fields of hematopoiesis and blood diseases (Figure 1), all of which are freely available without any registration. The majority of databases, namely Red Cell Membrane Disorder Mutations Database, dbRBC, CODEX, ErythronDB, Hembase, BloodSpot, and BloodChIP focus on the normal erythrocyte development in humans and model organisms to provide transcriptomic and genomic data. On the other hand, ELN and LGA are databases in the field of leukemia with clinical resources, whereas DBA mutation database is specifically designed for DBA. Obviously, despite our efforts on hematopoiesis studies, the sample sizes covered in the databases reviewed in this article are still limited and there is also lack of databases for other blood diseases. Fortunately, benefiting from big data programs across the globe, people are getting aware of the importance of biological data to public health, which makes it easier for researchers to obtain data generated from a large number of patients or donors. With the accumulation of knowledge and research progress, we are expecting to see a number of databases combined with clinical data available for biologists and clinicians in near future.

## Competing interests

The authors declared that there are no competing interests.

## Figures and Tables

**Table 1:** Main biological databases for hematology research

**Name**	**Weblink**	**Main features**	**Cell type**	**Data type**	**Refs.**
European LeukemiaNet	http://www.leukemia-net.org/content/home/index_eng.html	Providing physicians and patients research information about diagnosis, treatment, and ongoing clinical trials, as well as further information about leukemia	Clinical data of leukemia patients	Clinical data	[8]

Red Cell Membrane Disorder Mutations Database	http://research.nhgri.nih.gov/RBCmembrane	Grouping all mutation genes occurring in single or more kinds of inherited disorders of the erythrocyte membrane associated with hemolytic anemia	RBCs of hereditary spherocytosis, hereditary elliptocytosis, and hereditary pyropoikilocytosis patients	Mutation information of related genes	[9]

dbRBC	http://www.ncbi.nlm.nih.gov/projects/gv/rbc/main.fcgi?cmd=nit	Providing DNA and clinical data related to the human RBCs, integrated with BGMUT database documenting variations in genes that encode antigens for human blood groups	Human RBCs	DNA and clinical data	[10]

CODEX	http://codex.stemcells.cam.ac.uk	Containing a subunit database HAEMCODE specialized for grouping NGS data of human and mouse hematopoietic cell experiments	Human and mouse hematopoietic cells	NGS data	[12]

ErythronDB	http://www.cbil.upenn.edu/ErythronDB	Providing expression profile of murine primitive and definitive erythroid cells, and supporting gene searching with annotation, differential expression, transcriptional regulation, *etc.*	Murine primitive and definitive erythroid cells	Gene expression data	[13]

Hembase	http://hembase.niddk.nih.gov/	Integrating sequencing data of ESTs of human erythroid cells, differentiated erythrocytes, and mature RBCs	Human erythroid cells, differentiated erythrocytes, and mature RBCs	EST data	[1]

BloodSpot	http://www.bloodspot.eu	Providing gene expression profiles of healthy and malignant hematopoiesis in human or mice, encompassing more than 5000 samples in total	Human or mouse hematopoietic cells	Oligonucleotide microarray chip data and RNA-seq data	[15]

BloodChIP	http://www.med.unsw.edu.au/CRCWeb.nsf/page/BloodChIP	Exploring and visualizing TF sites in human CD34^+^ and other normal and leukemic cells based on TF ChIP-seq data	Human CD34^+^ and leukemic cells	Gene expression data, histone ChIP-seq data, DNase-seq data, and digital genomic footprinting data	[17]

Leukemia Gene Atlas	http://www.leukemia-gene-atlas.org/LGAtlas	Integrating datasets from more than 5800 leukemia and hematopoiesis samples sequenced by microarray, DNA methylation, SNP, and high-throughput sequencing	Clinical leukemia samples	Microarray, DNA methylation, and SNP data	[19]

DBA mutation database	http://www.dbagenes.unito.it	Integrating information on DBA mutation genes and changes of DNA, RNA, protein, and the frequency of the mutation	Blood cells of DBA	General information on variants of all DBA-related genes	[23], [24]

*Note*: ErythronDB, the Erythron Database; ChIP, chromatin immunoprecipitation; EST, expressed sequence tag; DBA, Diamond-Blackfan anemia; BGMUT, Blood Group Antigen Gene Mutation Database; NGS, next-generation sequencing; RBC, red blood cell; SNP, single nucleotide polymorphism; TF, transcription factor.
